# Comparative study between a spectral domain and a high-speed single-beam swept source OCTA system for identifying choroidal neovascularization in AMD

**DOI:** 10.1038/srep38132

**Published:** 2016-12-05

**Authors:** R. Told, L. Ginner, A. Hecht, S. Sacu, R. Leitgeb, A. Pollreisz, U. Schmidt-Erfurth

**Affiliations:** 1 Department of Ophthalmology and Optometry, Vienna Clinical Trial Center (vCTC), Medical University of Vienna, Vienna, Austria; 2 Center for Medical Physics and Biomedical Engineering, Medical University of Vienna, Vienna, Austria; 3 Christian Doppler Laboratory for Laser Development and their Application to Medicine and Biology, Center for Medical Physics and Biomedical Engineering, Medical University Vienna, Vienna, Austria

## Abstract

This comparative study between a SD- and SS-OCTA system for visualizing neovascular patterns in AMD, also assessed the influence of cataract on OCTA imaging. 25 eyes with active CNV (AMD) were documented by FA, ICGA and SD-OCT. Two OCTA devices were used: A custom built SS-OCTA (1050 nm, 400,000 A-scans/s, 5 × 5 mm, no image segmentation); AngioVue (OptoVue, CA, USA) SD-OCTA (840 nm, 70.000 A-scans/s, 3 × 3 mm, SSADA technology). Two retina experts graded CNV types and vascular patterns. Cataract influence on OCTA image quality was reported for the superficial retinal plexus (6 eyes). The SS-OCTA prototype showed more CNV lesions compared to the SD-OCTA system (p = 0.01). Overall sensitivity of SD- and SS-OCTA systems to detect CNV lesions was.32 and.68, respectively. The SS-OCTA system was able to detect discrete lesion characteristics better than the SD-OCTA. No significant difference was found in the ability to identify CNV in treatment-naïve eyes. There was no significant influence of cataract. The SS-OCTA prototype detected CNV-associated vascular patterns more reliably than the SD-OCTA system. This is attributed to the SS-OCTA system’s longer center wavelength and higher A-scan rate yielding higher definition and contrast of small neovascular structures. The SS-OCTA system used showed no advantage regarding cataract influence.

The introduction of optical coherence tomography (OCT)^1^,^2^ in 1991 has revolutionized retinal diagnostics in ophthalmology and has since evolved quickly. In particular, OCT imaging quality, speed and resolution have vastly improved over the last years. Today, the resolution of OCT enables visualization of cellular details at the photoreceptor level^3^.

A novel extension of the technology, OCT angiography (OCTA), allows non-invasive, dye- and contact-free detection of retinal and superficial choroidal blood flow and thus retinal and choroidal vasculature^4^,^5^. OCTA is based on the concept that in static tissue such as the neurosensory retina, the only dynamic structure is blood flow. Hence, visualizing flow-related changes reveals retinal and choroidal vasculature in high-resolution as well as a three-dimensional manner. OCTA was already shown to be a valuable tool for monitoring neovascular age-related macular degeneration (nAMD), as it clearly displays regression and recurrence/persistence of choroidal neovascularization (CNV) in distinct (sub-) retinal layers in response to anti-vascular endothelial growth factor (VEGF) treatment^6^,^7^.

Using longer wavelengths (~1050 nm) allows better tissue penetration^8^,^9^, as for example in the swept-source OCT angiography (SS-OCTA) system used in this study. Accordingly, OCTA systems based on 1 μm light sources offer improved immunity to ocular opacities such as cataract^10^. The resulting improved resolution of the choroid in these OCTA systems may also allow better visualization of CNV lesions, in particular occult CNV lesions and CNV-components spreading deep underneath the retinal pigment epithelium (RPE).

In this study, we compared volume scans of two different OCTA technologies in patients with nAMD. First, we used a commercially available spectral domain (SD-) OCTA system with 850 nm center wavelength (AngioVue^®^, Optovue, CA, USA) and second, a custom-built single-beam 1050 nm SS-OCTA for CNV lesion recording. In addition, the influence of cataract on image quality was assessed in recordings of both OCTA systems.

## Methods

The present study was performed in adherence to the Declaration of Helsinki including current revisions and the Good Clinical Practice (GCP) guidelines. The study protocol was approved by the Ethics Committee of the Medical University of Vienna. Scope and nature of this study were explained in detail to all subjects before they gave written informed consent. A total of 25 eyes with nAMD of 22 subjects were included in this comparative study.

Subjects were recruited at the Department of Ophthalmology and Optometry at the Medical University of Vienna, Austria. After assessing ETDRS best-corrected visual acuity (BCVA), a complete ophthalmic exam was performed including slitlamp examination, Goldman applanation tonometry, pupil dilation (Mydriaticum AGEPHA, Austria) and funduscopy. Patients eligible for study inclusion had to present with clear ocular media, except for 6 patients with cataract, and active neovascular AMD as assessed by fluorescein/indocyanin green angiography (FA/ICGA) and SD-OCT (Spectralis, Heidelberg Engineering, Germany). Finally, OCT angiography volumes of the CNV lesion using SD- and SS-OCTA systems were captured. Table one shows the characteristics of both SD- and SS-OCTA systems (Table 1).

### SS-OCTA system

**Figure 1:**
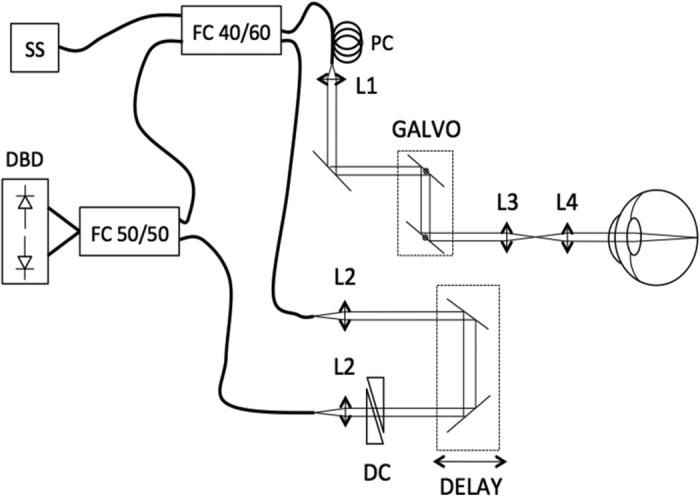
Schematic illustration of the SS-OCTA setup; SS: swept source, FC: fiber coupler, PC: polarization controller, DBD: dual balanced detector, DC: dispersion control, L1-L4: achromatic doublets.

Figure 1 shows the schematic setup of the SS-OCTA system^11^. The used light source (swept source, SS, AXSUN A13000467) operates at a 200 kHz sweep rate with a central wavelength of 1050 nm. The optical bandwidth is 110 nm with an axial resolution in tissue of 10 μm. The signal is measured with a dual-balanced detector (DBD, 1040392, Exalos). The total power at the cornea is ~1.2 mW, which is consistent with the ANSI standards safe exposure limits^12^, leading to a measured sensitivity of ~94 dB. The telescope (L3, L4) in the sample arm has an angular magnification of 1.5× and is used to change the focus within the eye. The beam size is ~1.3 mm at the cornea, corresponding to a calculated spot size of 25 μm on the retina.

The swept source laser consists of two multiplexed lasers, each operating at 100 kHz with a duty cycle of about 48% each. When the forward sweep (Sweep 1) of the first laser is finished, the corresponding back sweep is suppressed and the second laser starts the respective forward sweep (Sweep 2) and vice versa. Thus, the actual sweeping frequency is doubled to 200 kHz. To further increase the A-scan rate, intra-spectral sampling is used^11^. With the increased A-scan rate of 400 kHz a field of view of 16° can be obtained without image stitching. This equals area of 5 × 5 mm. OCTA is based on observing signal decorrelation due to moving red blood cells within scanned vessels. Therefore 4 B-scans at the same position are acquired. For OCTA processing, an adaptive threshold is applied to reduce motion artifacts^13^.

### SD-OCTA system

The SD-OCTA system used in this study is the commercially available AngioVue^®^ (Optovue, CA, USA) operating at a wavelength of 840 nm, acquiring 70.000 A-scans per second with an axial resolution of approximately 5 μm. OCTA volumes are derived from 2 consecutive orthogonal B-scans (2 × 304 × 304 A-scans). The systems native motion correction is performed online by co-registration of the two captured orthogonal volumes. Further, the split-spectrum amplitude-decorrelation algorithm (SSADA)^4^ is applied for improved signal-to-noise ratio. The system used allows recording OCTA volumes of 2 × 2 up to 6 × 6 mm. In order to assure maximum image resolution and coverage of the entire lesion, 3 × 3 mm volumes were used for OCTA comparison.

### OCTA C-Scan Grading

En face OCTA projections were used for best visualization of the neovascular complex. The AngioVue^®^’s native automated layer segmentation shows vascular architecture changes in the predefined superficial -, deep- and outer retina plexus as well as in the choroidal capillary layer. A retina expert manually corrected the accuracy of the implemented automated layer segmentation if necessary. A horizontal detection plane was used in SS-OCTA volumes for manual navigation through the retinal and choroidal layers for CNV grading. Corresponding OCT-B scans were used to guide placement and thickness of segmentation layers for optimal visualization of the CNV complex.

**Figure 2:**
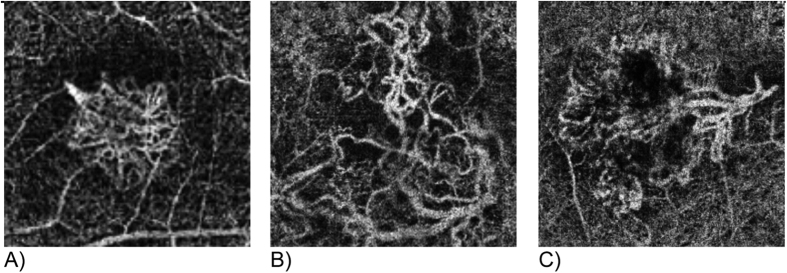
CNV types on SD-OCTA; (**A**) dense net: vascular net with branching capillaries; (**B**) vascular loop: loop-like configuration of multiple medium and larger size vessels; and (**C**) mixed CNV configuration: presence of both dense capillary net and vascular loop configuration in an individual CNV lesion. Images display a retinal area of approximately 3 × 3 mm.

Two retina experts independently graded CNV types and vascular patterns according to the following parameters: CNV types were defined as dense net, vascular loop, mixed configuration with both ‘dense net as well as vascular loop components present’, or no CNV complex visible (see Fig. 2).

**Figure 3:**
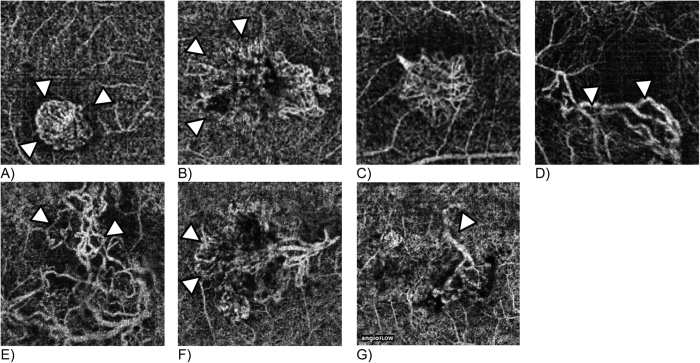
Examples of neovascular patterns on SD-OCTA: (**A**) well-defined lesion; (**B**) poorly-defined lesion; (**C**) branching capillaries; (**D**) mature vessels; (**E**) anastomoses; (**F**) peripheral arcading; (**G**) dead tree pattern; White arrows indicate areas of characteristic vascular changes. Images display a retinal area of approximately 3 × 3 mm.

The neovascular patterns included presence of a well-defined lesion, branching capillaries, mature vessels, anastomoses, peripheral arcading and dead tree pattern (see Fig. 3).

### Assessing the influence of cataract on image quality

Cataract was graded using the Lens Opacities Classification System III (LOCS scale)^14^. Image quality was assessed by comparing the microvascular structure of the superficial retinal plexus between the SD- and SS-OCTA systems. We hypothesized that cataract would decrease the fundus signal and therefore result in an artificial capillary drop-out. As no CNV might have been detectable in OCTA in the cataract subgroup, we graded OCTA volumes of the superficial retinal plexus for image quality: 0, if no microvasculature was present around the foveal avascular zone (FAZ); 1, if a non-perfused area around the FAZ appeared, and 2, if no change of the microvasculature around the FAZ was apparent.

### Statistics

Statistical analysis was performed using Prism 6 (SoftPad Software Inc. La Jolla, CA, USA). All data are reported as mean ± SD, median or as percent fraction of total. Chi-Square test and Fishers exact test were used to compare SD-OCTA and SS-OCTA measurements. Intraclass correlation coefficient (ICC) was calculated using SPSS 23 (IBM, USA) to estimate the agreement between two retinal experts (AP, RT). A two-way mixed model (absolute agreement) for single measures was used. P < 0.05 was considered the level of significance.

## Results

### Patients demographics

We enrolled 25 eyes of 22 patients with nAMD in this study. The mean age was 75 ± 8 years and 41% were male, 59% female. All patients met the common indication criteria for anti-VEGF treatment such as presence of intra- and/or subretinal fluid on OCT imaging. The presence of an underlying neovascular lesion in the course of AMD was confirmed by FA/ICGA. Thus, all patients had active exudative neovascular AMD. Six patients were treatment naïve, six patients presented with cataract.

### Intraclass correlation

Intraclass correlation (ICC) coefficient was used to estimate the agreement between individual measurements from readers one and two. ICC for CNV pattern grading (dense net, vascular loop, mixed configuration, no CNV) between the two retinal experts was greater than 97 indicating strong consistency of OCTA image grading. ICC for image quality due to cataract assessment was 1.

### OCTA imaging

Table 2 shows the results of the CNV lesion type grading for both, SD- and SS-OCTA volume scans. The prototype SS-OCTA system detected a higher CNV lesion rate for all lesion types including dense net, vascular loop and statistically significantly more mixed configurations (p = 0.04) than the SD-OCTA system. There was a statistically significant difference (p = 0.01) in the number of eyes where no CNV lesion could be detected: the used SS-OCTA failed to show a neovascular complex in 32% of eyes, whereas in the SD-OCTA 68% of eyes appeared to be CNV-free, despite a distinct presence of a neovascular lesion on FA/ICGA. This finding equals a sensitivity of .32 and .68 (n = 25) for the used SD- and SS-OCTA systems, respectively. In four treatment naïve nAMD eyes (66.7%, n = 6) no CNV associated pattern could be detected in SD-OCTA volumes. The SS-OCTA prototype failed to show a neovascular complex in three previously untreated eyes (50%). There was no significant difference between the two imaging approaches (p = 1, Fishers exact test) regarding presence of CNV lesions in treatment-naïve eyes.

A detailed morphologic CNV classification revealed heterogeneous neovascular patterns (see Table 3). However, the SS-OCTA approach was able to detect CNV patterns involving discrete details such as branching capillaries (n = 11/6, SS- and SD-OCT, respectively), anastomoses (9/5) and well-defined CNV lesions (6/5), more often than the SD-OCTA system. Yet, this did not reach the level of significance.

### Influence of cataract on image quality

Median LOCS scale^14^ of 6 eyes was NII, PII, CI. There was no significant difference between SD- and SS-OCTA regarding the ability to identify the superficial retinal plexus. None of the SD- and SS-OCTA volumes was graded zero or one. Accordingly, cataract was not found to have a significant influence on OCTA image quality in both, SD- and SS-OCTA systems.

## Discussion

OCTA is a relatively new imaging technology, which allows non-invasive visualization of retinal and choroidal vessels by capturing their perfusion^4^,^11^. The ability to visualize and monitor neovascular changes^6^,^7^ occurring in active AMD disease is the reason why OCTA is more and more frequently used.

This comparative study of a commercially available SD-OCTA system and a prototype SS-OCTA in eyes with nAMD is of clinical relevance, since a precise and simultaneous visualization of different retinal and choroidal vascular layers is a prerequisite for understanding disease pathogenesis and definition of future CNV retreatment and therapy response criteria. This would be analogous to morphological findings in OCT B-scans such as intraretinal cystoid spaces, subretinal fluid or pigment-epithelial detachments^15^, which are used daily in clinical routine.

**Figure 4:**
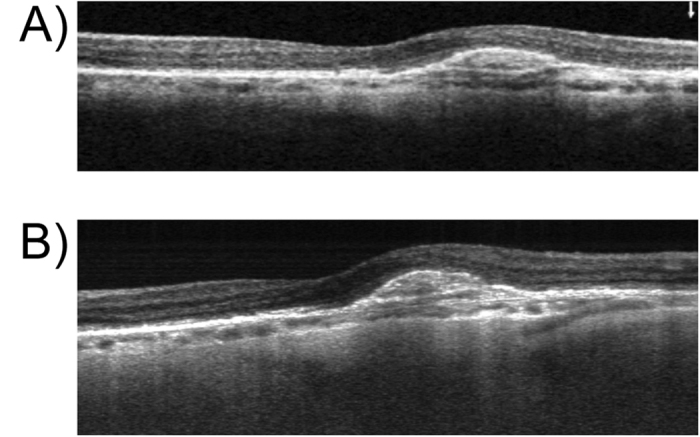
Exemplary 3 mm B-scans acquired with SD-OCTA (**A**) and SS-OCTA (**B**) systems. The AngioVue^®^ (OptoVue, CA, USA) SD-OCTA displays averaged B-scans consisting of two individual scans. The prototype SS-OCTA system used in this study records four B-scans for each position of the OCTA volume, which are displayed as an average B-scan in B. Lesion details, particularly in respect to deeper layers, appear more distinctly on SS-OCTA images.

Recent studies indicate equal performance of 840 nm SD- and 1050 nm SS-OCT regarding the visualization of specific morphologic landmarks such as the choroidal-scleral interface^16^. However, improved image quality of 1050 nm compared to 840 nm frequency domain OCT in patients with retinal pathologies and coexisting cataract was previously shown^10^. As outlined earlier, superior tissue penetration of long-wavelength SS-OCT compared to SD-OCT using enhanced depth imaging was reported^17^. This can be appreciated in Fig. 4, showing the superior tissue penetration of the SS-OCTA system compared to the SD-OCTA system used in this study.

In OCTA, increasing the A-scan rate from 70 to 400 kHz allows extending the field of view, while maintaining the same lateral sampling and thus keeping high microvascular contrast and signal to noise ratio.

In this study, we hypothesized that the used SS-OCTA setup without additional processing efforts is able to better visualize CNV lesions compared to a commercially available SD-OCTA and further that this is also true in patients with media opacities, in particular cataract.

Novais *et al*. recently reported significantly larger CNV demarcation areas in a similar approach as we used in this study, comparing a 400 kHz SS-OCTA to a commercially available 70 kHz SD-OCTA^18^. Our findings confirm these earlier results, as small lesion details such as branching capillaries, anastomoses or peripheral arcades were more frequently detected in our SS-OCTA than in SD-OCTA recordings. Also, the number of OCTA volumes, which did not contain any signs of choroidal neovascularization was significantly higher with the SD-OCTA system than with the SS-OCTA setup used. These findings illustrate that SD-OCTA misses flow information, especially of micro vessels, despite layer segmentation and SSADA^4^ processing.

A sensitivity of 50% and specificity of 91% for detecting CNV by the same SD-OCTA system we used in our study was reported^19^. Gong *et al*. report a sensitivity of 87% and a specificity of 68% for the detection of CNV also using the same commercially available SD-OCTA system (AngioVue^®^, OptoVue, CA, USA)^20^. This study shows the sensitivity of SD-OCTA being as low as 32%. The SS-OCTA prototype had a sensitivity of 68%, which highlights its superior ability for CNV detection.

We further hypothesized that there might be a difference between SD- and SS-OCTA setups regarding the ability to capture a CNV complex in treatment-naïve eyes. However, there was no significant difference between both technologies. This might in part be due to the high amount of fluid present before the application of an anti-VEGF loading dose and the resulting signal attenuation as well as fluid-induced difficulties in retinal layer segmentation due to loss of backscattering contrast between layers, which applies to both technologies. Biological fluid contains scattering constituents that lead to attenuation of light. The amount of attenuation is determined by the fluid density and the probing wavelength. Longer wavelengths such as 1050 nm as compared to 840 nm are expected to be scattered less, leading in general to better contrast below such fluid layers. This may be an explanation for the low sensitivity of SD- and SS-OCTA systems in this study as compared to previous reports^19^,^20^. It has to be kept in mind that both previous studies are retrospective and one does not indicate whether treatment naïve patients were included. This could lead to a selection bias and therefore higher sensitivity, as opposed to this prospective study.

**Figure 5:**
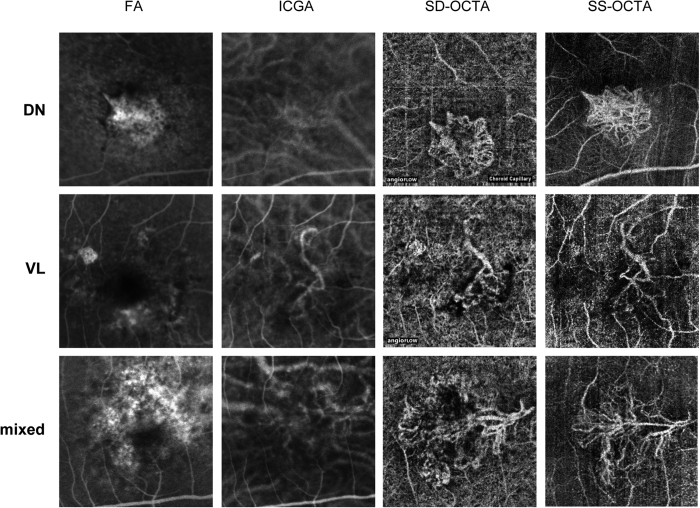
Examples of characteristic neovascular patterns: dense net (DN), vascular loop (VL), and mixed configuration. CNV lesions were imaged by FA, ICGA, SD-OCTA and SS-OCTA (maximum intensity projections). The images displayed equal the 3 × 3 mm area of the SD-OCTA system.

On direct comparison with FA/ICGA, the superior ability of OCTA to illustrate blood flow and thus detailed vessel configuration in a neovascular complex can be appreciated (Fig. 5), especially when it comes to analyzing specific retinal and choroidal layers. Inoue *et al*. concluded that in patients with type 1 nAMD, SD-OCTA (AngioVue^®^, OptoVue, CA, USA) combined with structural OCT is superior to FA alone in detecting type 1 nAMD lesions^21^. In another study assessing the diagnostic accuracy of SD-OCTA (AngioVue^®^, OptoVue, CA, USA) and FA, OCTA detected more eyes (56 eyes) with nAMD compared to FA alone (52 eyes)^20^. However, due to the lack of new clinical guidelines, FA/ICGA are still considered the gold standard in nAMD diagnosis^22^.

Currently, the resolution of OCTA images is of limited clinical importance in exudative AMD, since a combination of clinical examination, presence of vascular alterations in FA/ICGA and morphologic parameters on conventional OCT triggers the decision for intravitreal ocular medication. Even though OCTA-guided CNV retreatment criteria are not yet established, it is likely a matter of time until OCTA is fully integrated in the personalized management of AMD. However, better tissue penetration, increased visibility of microvascular structures and an expanded field of view as achieved by the used SS-OCTA prototype may be of relevance in order to fully visualize the extent of the CNV lesion when investigating patients with nAMD, in particular patients with occult type 1 CNV, which is located beneath the RPE layer leading to signal attenuation^17^,^23^ or in patients with media opacities. Further, microvessels captured with the SD-OCTA system appear thicker than in our prototype SS-OCTA, which might be due to different lateral spot sizes of the systems or to the application of a smoothing filter such as a Gaussian filter in post-processing in the case of SD-OCTA. This difference may be of interest in the future, once CNV retreatment criteria will be based on OCTA. Considering the fast technical advances in this innovative field of research, high-resolution and depth-resolved OCTA volumes of neovascular lesions with integrated en-face information on the exudative status of the lesion^15^,^24^ might soon become available and may replace invasive and time-consuming FA/ICGA^25^, which bares potential risks such as allergic shock^26^. This combination would allow early detection, as well as visualization and precise monitoring of the full CNV lesion in the course of the disease. Therefore, future approaches defining retreatment criteria based on OCTA will surely benefit from high-resolution OCTA systems, such as the one used in this study^11^, as a commercially available 200 kHz light source is used and enhanced to 400 kHz by applying intra-spectral sampling.

The small sample size of this study has to be considered a limitation. This is especially true for the cataract and treatment-naïve subgroup. However, we could not detect any significant differences in image quality whatsoever between the SS- and SD-OCTA systems used in patients with exudative AMD and cataract in this population. Further studies investigating the influence of cataract are warranted as the previously described superior ability of swept source OCT in penetrating cataract might only become evident in more mature cataracts. A limitation of OCTA technology is due to projection artifacts, making the interpretation for non-expert readers challenging. Furthermore, not all CNV complexes are visualized on OCTA, which can be due to imperfect layer segmentation or for example fluid-induced loss of backscattering contrast between layers. Manual correction of retinal layer segmentation is required for accurate interpretation.

In conclusion this study, which was carried out in a prospective manner following a protocol including FA/ICGA and SD-OCT for nAMD CNV detection, showed that the used SS-OCTA setup detects neovascular lesions and its patterns more reliably than the SD-OCTA device using manually corrected automated layer segmentation and the SSADA algorithm. However, no significant influence of cataract on both, SD- and SS-OCTA image quality was found. Denser lateral sampling of the SS-OCTA system, which is achieved by increasing the A-scan rate to 400 kHz, allows better visualization of vascular details and using a higher A-scan rate allows scanning a larger field of view without loosing vascular detail.

## Additional Information

**How to cite this article**: Told, R. *et al*. Comparative study between a spectral domain and a high-speed single-beam swept source OCTA system for identifying choroidal neovascularization in AMD. *Sci. Rep.* **6**, 38132; doi: 10.1038/srep38132 (2016).

**Publisher's note:** Springer Nature remains neutral with regard to jurisdictional claims in published maps and institutional affiliations.

## Figures and Tables

**Table 1:** OCTA system characteristics.

	SD-OCTA	SS-OCTA
OCTA	spectral domain	swept source
Wavelength	~840 nm	~1050 nm
A-scans per second	70,000	400,000
Total A-Scans	2 × 304 × 304	4 × 1600 × 400
B-scan repetition	2	4
Acquisition time	3 sec.	6.7 sec.
Axial resolution	5 μm	10 μm

**Table 2:** Summary of CNV lesion types as assessed by SD- and SS-OCTA systems; n = 25.

	SD-OCTA [%]	SS-OCTA [%]	p-value*
dense net	16	24	0.48
vascular loop	12	20	0.44
mixed configuration	4	24	0.04
no CNV	68	32	0.01

^*^Chi-Square test.

**Table 3:** Summary of neovascular patterns as assessed by SD- and SS-OCTA systems; n = 25.

	SD-OCTA [%]	SS-OCTA [%]	p-value*
Well-defined lesion	20	24	0.9
Branching tiny capillaries	24	44	0.6
Rare large vessels	16	36	0.9
Anastomoses	20	36	0.7
Peripheral arcade	4	8	0.9
Peripheral dead tree	4	12	0.7

^*^Chi-Square test.

## References

[b1] Fercher A. F., Hitzenberger C. K., Drexler W., Kamp G. & Sattmann H. *In vivo* optical coherence tomography. Am J Ophthalmol 116, 113–114 (1993). 8328536 10.1016/s0002-9394(14)71762-3

[b2] Huang D. . Optical coherence tomography. Science 254, 1178–1181 (1991). 1957169 10.1126/science.1957169 PMC4638169

[b3] Felberer F. . Adaptive optics SLO/OCT for 3D imaging of human photoreceptors *in vivo*. Biomed Opt Express 5, 439–456, doi: 10.1364/BOE.5.000439 (2014). 24575339 PMC3920875

[b4] Jia Y. . Split-spectrum amplitude-decorrelation angiography with optical coherence tomography. Opt Express 20, 4710–4725, doi: 10.1364/OE.20.004710 (2012). 22418228 PMC3381646

[b5] Zotter S. . Visualization of microvasculature by dual-beam phase-resolved Doppler optical coherence tomography. Opt Express 19, 1217–1227, doi: 10.1364/OE.19.001217 (2011). 21263663 PMC3036955

[b6] Huang D., Jia Y., Rispoli M., Tan O. & Lumbroso B. Optical Coherence Tomography Angiography of Time Course of Choroidal Neovascularization in Response to Anti-Angiogenic Treatment. Retina 35, 2260–2264, doi: 10.1097/IAE.0000000000000846 (2015). 26469535 PMC4627371

[b7] Coscas, G. . Optical coherence tomography angiography during follow-up: qualitative and quantitative analysis of mixed type I and II choroidal neovascularization after vascular endothelial growth factor trap therapy. Ophthalmic Res 54, 57–63, doi: 10.1159/000433547 (2015). 26201877

[b8] Unterhuber A. . *In vivo* retinal optical coherence tomography at 1040 nm - enhanced penetration into the choroid. Opt Express 13, 3252–3258 (2005). 19495226 10.1364/opex.13.003252

[b9] Yasuno Y. . *In vivo* high-contrast imaging of deep posterior eye by 1-microm swept source optical coherence tomography and scattering optical coherence angiography. Opt Express 15, 6121–6139 (2007). 19546917 10.1364/oe.15.006121

[b10] Povazay B. . Three-dimensional optical coherence tomography at 1050 nm versus 800 nm in retinal pathologies: enhanced performance and choroidal penetration in cataract patients. J Biomed Opt 12, 041211, doi: 10.1117/1.2773728 (2007). 17867800

[b11] Ginner L. . Wide-Field OCT Angiography at 400 KHz Utilizing Spectral Splitting. Photonics 1, 369–379 (2014).

[b12] ANSI. In American National Standard for Safe Use of Lasers Vol. ANSI Z136.1-2000 (American National Standards Institute, 2000).

[b13] Blatter C. . Dove prism based rotating dual beam bidirectional Doppler OCT. Biomedical optics express 4, 1188–1203, doi: 10.1364/BOE.4.001188 (2013). 23847742 PMC3704098

[b14] Chylack L. T. Jr. . The Lens Opacities Classification System III. The Longitudinal Study of Cataract Study Group. Arch Ophthalmol 111, 831–836 (1993). 8512486 10.1001/archopht.1993.01090060119035

[b15] Waldstein S. M. . Predictive Value of Retinal Morphology for Visual Acuity Outcomes of Different Ranibizumab Treatment Regimens for Neovascular AMD. Ophthalmology 123, 60–69, doi: 10.1016/j.ophtha.2015.09.013 (2016). 26481821

[b16] Philip A. M. . Choroidal thickness maps from spectral domain and swept source optical coherence tomography: algorithmic versus ground truth annotation. Br J Ophthalmol 100, 1372–1376, doi: 10.1136/bjophthalmol-2015-307985 (2016). 26769670 PMC5774332

[b17] Adhi M. . Enhanced visualization of the choroido-scleral interface using swept-source OCT. Ophthalmic Surg Lasers Imaging Retina 44, S40–42, doi: 10.3928/23258160-20131101-08 (2013). 24220884

[b18] Novais E. A. . Choroidal Neovascularization Analyzed on Ultrahigh-Speed Swept-Source Optical Coherence Tomography Angiography Compared to Spectral-Domain Optical Coherence Tomography Angiography. Am J Ophthalmol 164, 80–88, doi: 10.1016/j.ajo.2016.01.011 (2016). 26851725 PMC4811690

[b19] de Carlo T. E. . Spectral-domain optical coherence tomography angiography of choroidal neovascularization. Ophthalmology 122, 1228–1238, doi: 10.1016/j.ophtha.2015.01.029 (2015). 25795476

[b20] Gong J., Yu S., Gong Y., Wang F. & Sun X. The Diagnostic Accuracy of Optical Coherence Tomography Angiography for Neovascular Age-Related Macular Degeneration: A Comparison with Fundus Fluorescein Angiography. J Ophthalmol 2016, 7521478, doi: 10.1155/2016/7521478 (2016). 27110394 PMC4821972

[b21] Inoue, M. . A Comparison Between Optical Coherence Tomography Angiography and Fluorescein Angiography for the Imaging of Type 1 Neovascularization. Invest Ophthalmol Vis Sci 57, OCT314-323, doi: 10.1167/iovs.15-18900 (2016). 27409488

[b22] Schmidt-Erfurth U. . Guidelines for the management of neovascular age-related macular degeneration by the European Society of Retina Specialists (EURETINA). Br J Ophthalmol 98, 1144–1167, doi: 10.1136/bjophthalmol-2014-305702 (2014). 25136079 PMC4145443

[b23] Sayanagi K. . En-face high-penetration optical coherence tomography imaging in polypoidal choroidal vasculopathy. Br J Ophthalmol 99, 29–35, doi: 10.1136/bjophthalmol-2013-304658 (2015). 25107899

[b24] Waldstein S. M. . Correlation of 3-Dimensionally Quantified Intraretinal and Subretinal Fluid With Visual Acuity in Neovascular Age-Related Macular Degeneration. JAMA ophthalmology 134, 182–190, doi: 10.1001/jamaophthalmol.2015.4948 (2016). 26661463

[b25] Laatikainen L. The fluorescein angiography revolution: a breakthrough with sustained impact. Acta Ophthalmol Scand 82, 381–392, doi: 10.1111/j.1395-3907.2004.00284.x (2004). 15291929

[b26] Ha S. O., Kim D. Y., Sohn C. H. & Lim K. S. Anaphylaxis caused by intravenous fluorescein: clinical characteristics and review of literature. Intern Emerg Med 9, 325–330, doi: 10.1007/s11739-013-1019-6 (2014). 24293212

